# HINT1 protein cooperates with cannabinoid 1 receptor to negatively regulate glutamate NMDA receptor activity

**DOI:** 10.1186/1756-6606-6-42

**Published:** 2013-10-05

**Authors:** Ana Vicente-Sánchez, Pilar Sánchez-Blázquez, María Rodríguez-Muñoz, Javier Garzón

**Affiliations:** 1 Neuropharmacology, Instituto Cajal, CSIC, Madrid E-28002, Spain

**Keywords:** HINT1 protein, Cannabinoid receptor, NMDA receptor, Cortical neuron cultures, Excitotoxicity, Neuroprotection

## Abstract

**Background:**

G protein-coupled receptors (GPCRs) are the targets of a large number of drugs currently in therapeutic use. Likewise, the glutamate ionotropic *N*-methyl-D-aspartate receptor (NMDAR) has been implicated in certain neurological disorders, such as neurodegeration, neuropathic pain and mood disorders, as well as psychosis and schizophrenia. Thus, there is now an important need to characterize the interactions between GPCRs and NMDARs. Indeed, these interactions can produce distinct effects, and whereas the activation of Mu-opioid receptor (MOR) increases the calcium fluxes associated to NMDARs, that of type 1 cannabinoid receptor (CNR1) antagonizes their permeation. Notably, a series of proteins interact with these receptors affecting their responses and interactions, and then emerge as novel therapeutic targets for the aforementioned pathologies.

**Results:**

We found that in the presence of GPCRs, the HINT1 protein influences the activity of NMDARs, whereby NMDAR activation was enhanced in CNR1^+/+^/HINT1^-/-^ cortical neurons and the cannabinoid agonist WIN55,212-2 provided these cells with no protection against a NMDA insult. NMDAR activity was normalized in these cells by the lentiviral expression of HINT1, which also restored the neuroprotection mediated by cannabinoids. NMDAR activity was also enhanced in CNR1^-/-^/HINT1^+/+^ neurons, although this activity was dampened by the expression of GPCRs like the MOR, CNR1 or serotonin 1A (5HT1AR).

**Conclusions:**

The HINT1 protein plays an essential role in the GPCR-NMDAR connection. In the absence of receptor activation, GPCRs collaborate with HINT1 proteins to negatively control NMDAR activity. When activated, most GPCRs release the control of HINT1 and NMDAR responsiveness is enhanced. However, cannabinoids that act through CNR1 maintain the negative control of HINT1 on NMDAR function and their protection against glutamate excitotoxic insult persists.

## Background

The glutamatergic *N*-methyl-D-aspartate (NMDA) receptor has a significant influence on the efficacy of neurotransmission and synaptic plasticity, and on processes such as learning and memory. The activation of this ionotropic receptor results in the permeation of Ca^2+^ ions, and it is positively regulated by certain G protein-coupled receptors (GPCRs) through the activity of PKC and Src [1,2]. Whilst, the Mu-opioid receptor (MOR) interacts with the NMDAR [3] positively regulating NMDAR calcium fluxes [4], the cannabinoid 1 receptor (CNR1) dampens the activity of this glutamate ionotropic receptor [5]. Notably, the regulation of MORs and of CNR1s on NMDARs activity requires the histidine triad nucleotide-binding protein 1 (HINT1), and in its absence these interactions are weakened to the extent that morphine no longer stimulates NMDAR activity and WIN55,212-2 fails to inhibit it [5-7].

The activation of CNR1s in the presynapse reduces the release of glutamate into the cleft and contributes to NMDAR hypofunction [8]. Nevertheless, CNR1s in the postsynapse also negatively regulate NMDAR function by interfering with its signaling pathways [9]. However, cannabinoids prevent the endogenous increase of calcium through mechanisms related to the direct inhibition of NMDAR calcium influx [9,10], as also suggested using whole-cell patch clamp recording techniques [11]. This mechanism would account for cannabinoid control of exogenous activators of NMDAR function. Thus, besides interacting with distant signaling pathways, cannabinoids can also directly affect the open probability of the NMDAR calcium channel. Immunocytochemical and ultrastructural studies demonstrated the presence of CNR1s in the post-synapse at both the spinal [12-14] and supraspinal level [15,16], where they co-localize with NMDARs and PSD95 proteins [5,17]. Indeed, co-immunoprecipitation assays performed ex vivo on mouse cerebral cortical synaptosomes and in vitro studies with recombinant proteins revealed the association between CNR1s and NMDARs [5,18]. In the context of CNR1-NMDAR association, cannabinoids disassemble and inactivate CNR1-associated NMDARs through the co-internalization of NR1 subunits [5], and probably of surface NMDAR NR2 subunits as well [19].

HINT1 is a 126 amino acid protein of approximately 14 kDa that belongs to the histidine triad (HIT) family, all members of which contain the HisXHisXHis sequence (where X is any hydrophobic amino acid). HINT1 binds zinc and purine nucleotides, and while it exists as a zinc-independent homodimer, zinc ions mediate the interaction between HINT1 and third party proteins [6,20,21]. This protein was initially referred to as a protein kinase C-interacting protein (PKCi) as it binds to and inhibits PKC function in a zinc-dependent manner [22]. However, this activity has since been relegated in favor of its enzymatic activity [23]. Notwithstanding, HINT1 appears to be implicated in a wide variety of physiological processes, some of these functions are independent of HINT1 enzymatic activity [24], such as DNA damage response pathways and tumor suppression pathways [25], repression of β-catenin signaling and transcriptional regulation [26], and regulation of cell endogenous calcium signaling [27]. Furthermore, there is evidence that at the plasma membrane HINT1 regulates GPCR function via PKC [7,28-31], and that it regulates the interaction of NMDARs with GPCRs like the MOR and CNR1 [5,6]. At the cell membrane, HINT1 exists as a homodimer and it behaves as a scaffold protein regulated by Redox processes that bring together different signaling pathways under the regulation of GPCRs [32,33]. In this way it is possible to reconcile the enzymatic activity of the HINT1 protein with its role as a switch in conveying information mediated by GPCR to different signaling pathways, e.g., glutamate NMDAR-mediated synaptic plasticity, β-catenin regulation, calcium signaling and DNA repair.

HINT1 associates directly with the cytosolic regions of NMDAR NR1 subunits and with those of certain GPCRs, recruiting a series of signaling proteins to the receptor environment [5,30-32]. Indeed, the cytosolic C terminal sequence of the MOR, and also that of CNR1, bind the cytosolic C1 segment of the NMDAR NR1 subunit, and HINT1 stabilizes this association [5,6]. Morphine challenge promotes the assembly and dis-assembly a series of signaling proteins at the MOR-HINT1 complex in order to enhance the activity of the NMDAR [30,31], which in turn negatively controls the effects of opioids [34,35]. The activation of the CNR1 has the opposite effect and it diminishes NMDAR activity. The negative regulation of NMDARs is particularly relevant because overactivation of NMDARs produces a series of perturbations that are associated with neurodegenerative diseases [36], mood disorders such as schizophrenia and depression [37,38], and neuropathic pain [39,40]. Indeed, cannabinoids are highly efficient in reducing calcium permeation mediated by NMDARs and in protecting neurons from the potential risks of excessive NMDAR activity [41,42]. Cannabinoid receptors are distributed throughout the central nervous system [43] and the CNR1 is present at high densities in presynaptic terminals [44], as well as in postsynaptic structures of spinal and supraspinal glutamatergic synapses [12,14,15]. As the HINT1 protein associates with the cytosolic regions of CNR1 [5,18,31], we sought to determine the effects of HINT1 on the cannabinoid-mediated negative control of glutamate NMDAR function. Our study indicates that in the absence of receptor activation, different GPCRs, including CNR1, collaborate with HINT1 proteins to reduce the sensitivity of NMDARs to stimulation. However, upon GPCR activation, cannabinoids maintain HINT1-mediated control over NMDAR activity and they protect against NMDA-associated excitotoxicity.

## Results

### Increased NMDA-induced neuronal injury and the absence of cannabinoid neuroprotection in HINT1^-/-^ cultured cortical neurons

**Figure 1:**
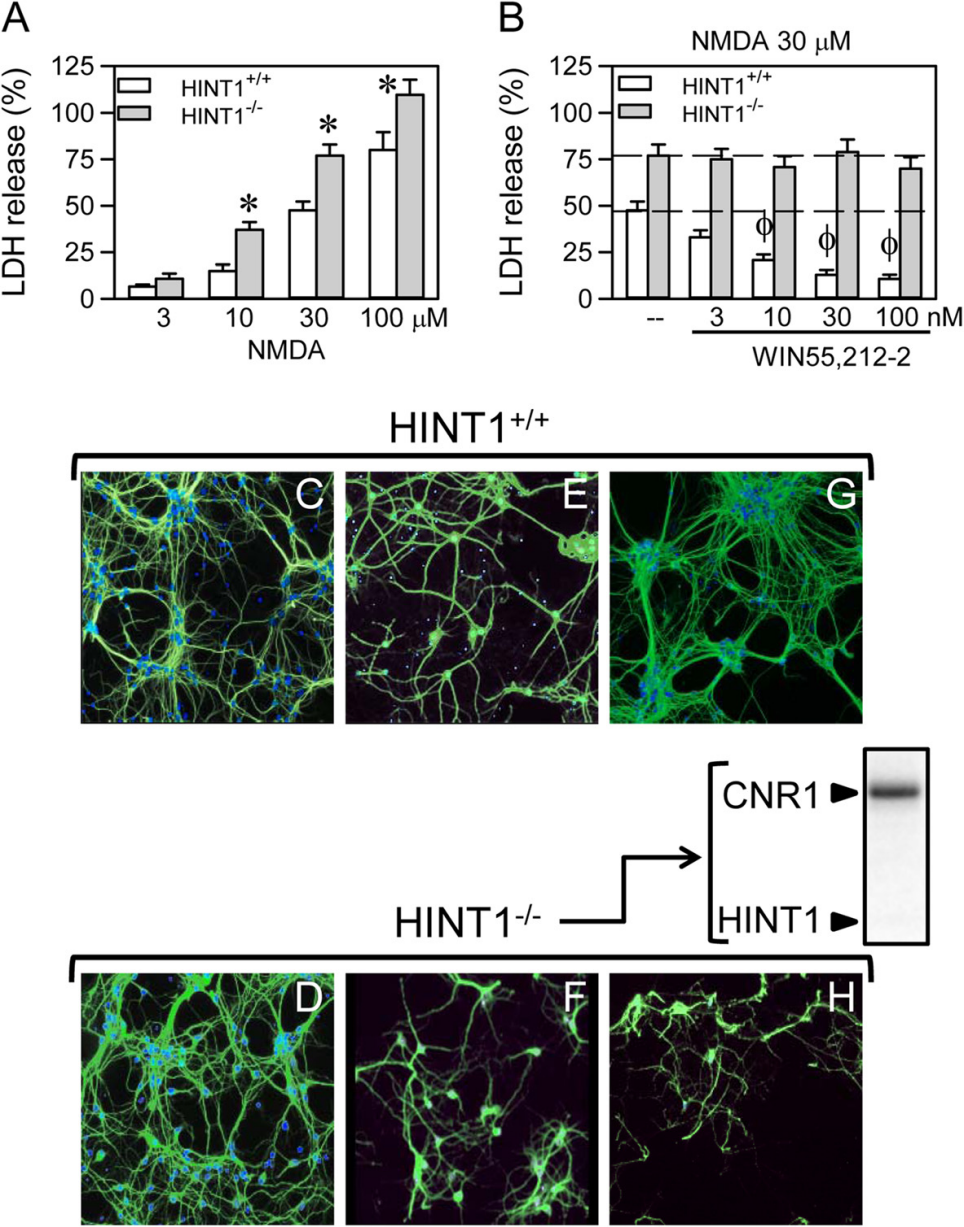
**NMDA-induced neuronal injury in cortical cell cultures from WT and HINT1**^**-/- **^**mice*. *(A)** Cultures were exposed to increasing concentrations of NMDA for 24 h. Cell death was measured by LDH efflux into the medium. The data shown represent the mean ± S.E.M. from 20 wells per group. * Significant difference between WT and HINT1^-/-^ cultured neurons, *p* < 0.05. **(B)** The cultures were exposed to a fixed concentration of 30 μM NMDA for 24 h in the absence (--) or presence of increasing concentrations of the cannabinoid agonist WIN55,212-2. To eliminate the possible participation of cannabinoid receptor 2 (CNR2), the assay was conducted in the presence of 3 μM of the CNR2 antagonist JTE907. Φ Significant difference compared to NMDA alone, *p* < 0.05. **(C**-**H)** Fluorescence photomicrograph of cortical cell cultures immunolabelled with an anti-MAP2 antibody (Ab) (green) following treatment with vehicle **(C** and **D)**, 30 μM NMDA **(E** and **F)** or 30 μM NMDA plus 100 nM WIN55,212-2 **(G** and **H)**; the nuclei were counterstained with 4,6-diamidino-2-phenylindole. Inset: Immunodetection study of CNR1 (antibody C terminal sequence 461–472; Cayman Chemical, Mi, USA, 10006590) and HINT1 (antibody from Abnova H00003094-A01. Abyntek, Spain) in HINT1^-/-^ cultured neurons (40 μg/lane).

The exposure of neuronal-enriched E16 murine cortical cultures from wild-type (WT) (HINT1^+/+^) and HINT1^-/-^ mice to NMDA for 24 h resulted in a concentration-dependent decrease in cell viability, as measured by LDH release. In all cases, NMDA toxicity was greater in the knockout cells (Figure 1A,E,F). Pre-treatment or co-treatment with the cannabinoid agonist WIN55,212-2 reduced the toxicity of NMDA (30 μM) in the WT cultures (Figure 1B,E,G). Furthermore, the effects of WIN55,212-2 against NMDA insult were abrogated upon treatment with the CNR1 antagonist SR141716A (1 μM), which is consistent with CNR1-mediated neuroprotection. However, in the absence of HINT1, the positive effect of the cannabinoid agonist was absent (Figure 1B,F,H).

**Figure 2:**
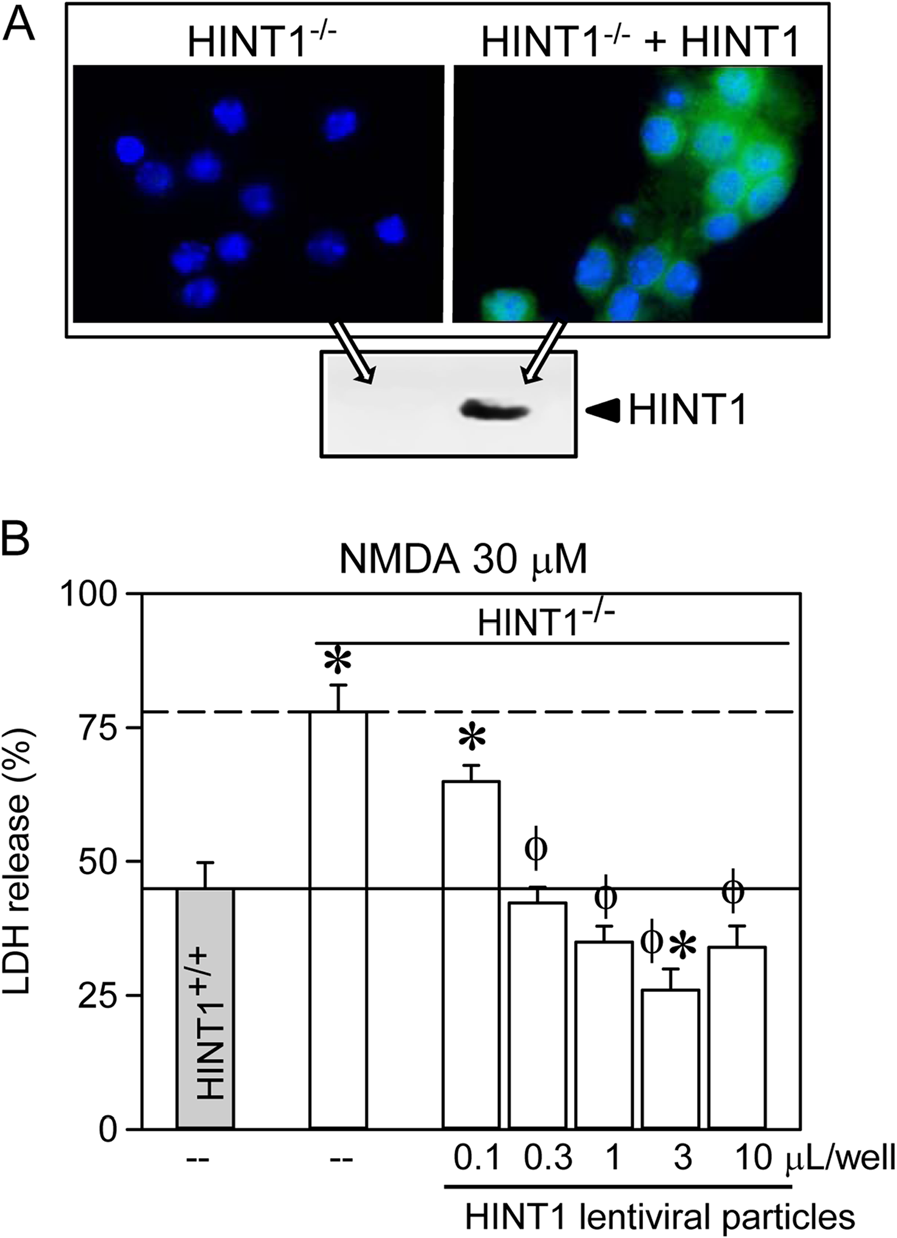
**Lentiviral expression of HINT1 increases cell viability in the presence of NMDA. (A)** Fluorescence photomicrograph of HINT1^-/-^ cortical cell cultures uninfected or infected with lentiviral particles. The cells were immunolabelled with anti-HINT1 Ab (green). The nuclei were counterstained with 4,6-diamidino-2-phenylindole. Inset: Immunodetection of HINT1. **(B)** The cultures were infected and exposed to 30 μM of NMDA for 24 h. Cell death was measured by LDH efflux into the medium. The data shown represent the mean ± S.E.M. from 20 wells per experimental group. * Indicates a significant difference between HINT1^-/-^ cortical cell cultures, *p* < 0.05, compared to those derived from WT mice. Φ Significant increase in cell viability of HINT1^-/-^ cortical cell cultures infected with increasing amounts of the viral HINT1 vector compared to the HINT1^-/-^ control group, *p* < 0.05.

**Figure 3:**
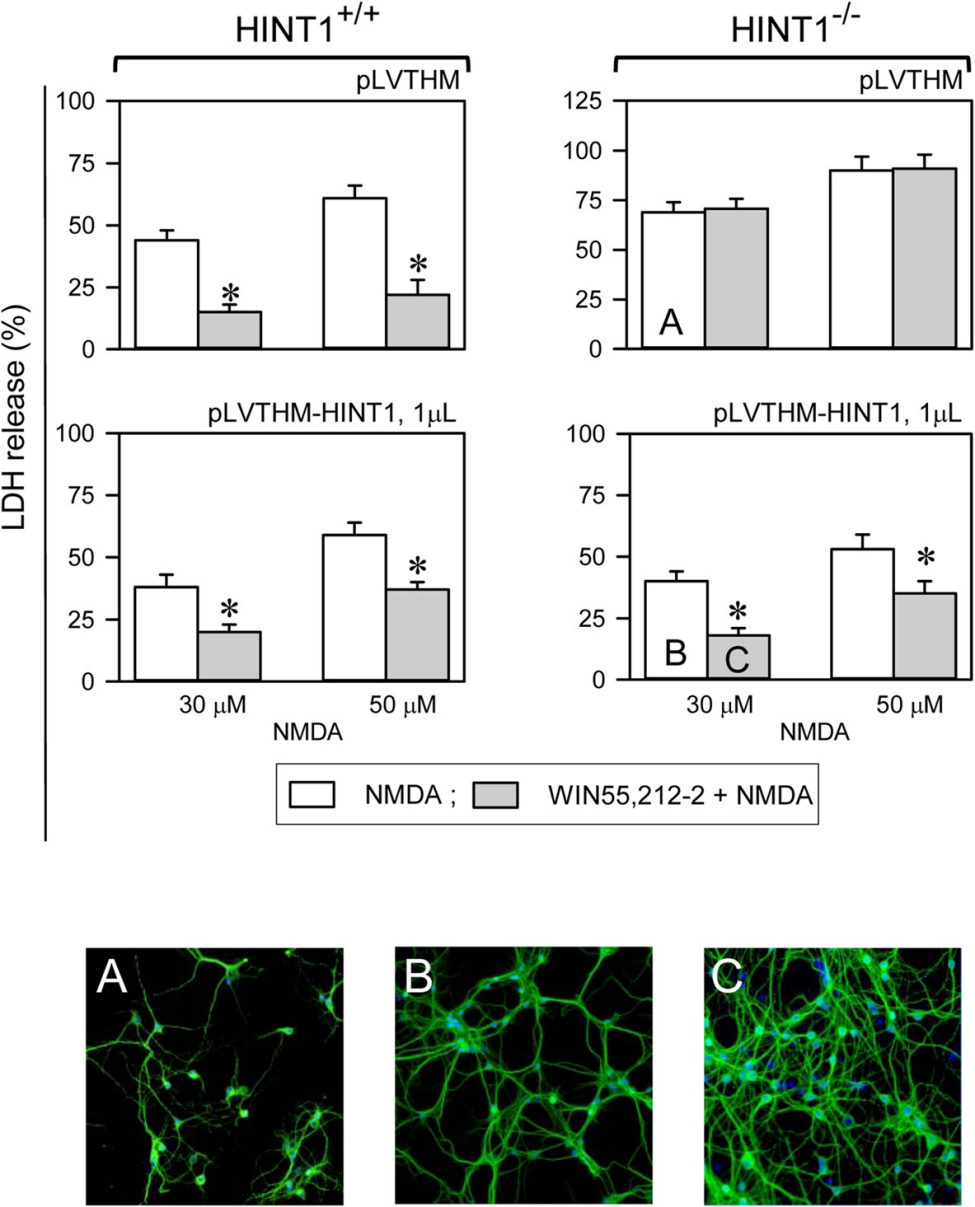
**The effect of lentiviral expression of HINT1 on WIN55,212-2-mediated neuroprotection against NMDA insult.** The cell cultures from WT and HINT1^-/-^ mice were infected with 0.1 or 3 μL of lentiviral particles and exposed to NMDA for 24 h in the absence or presence of 100 nM of WIN55,212-2. Cell death was measured by LDH efflux into the medium. The data shown represent the mean ± S.E.M. from 20 wells per condition. * Significant difference compared to NMDA alone, *p* < 0.05. Bottom: Fluorescence photomicrographs of NMDA-treated HINT1^-/-^ cortical cell cultures **(A)** uninfected or infected with HINT1 lentiviral particles either **(B)** without or **(C)** with WIN55,212-2. The cells were immunolabelled with an anti-MAP2 Ab (green). The nuclei were counterstained with 4,6-diamidino-2-phenylindole.

We then infected HINT1^-/-^ cortical cultures with lentiviral vectors expressing the HINT1 protein. Transgene expression was significantly detected 3 days post-transduction (Figure 2A). After NMDA exposure for 24 h (30 μM), the viability of the HINT1^-/-^ neurons transduced with pLVTHM-HINT1 (0.1 to 3 μL/well lentiviral particles) was greater than the viability of pLVTHM-transduced HINT1^-/-^ neurons and WT HINT1^+/+^ neurons (Figure 2B). Infection of HINT1^-/-^ neurons with 1 μL/well of the lentiviral particles also restored WIN55,212-2-mediated neuroprotection against NMDA toxicity (Figure 3A,B,C).

### The enhanced response of HINT1^-/-^ neurons to NMDAR activation

**Figure 4:**
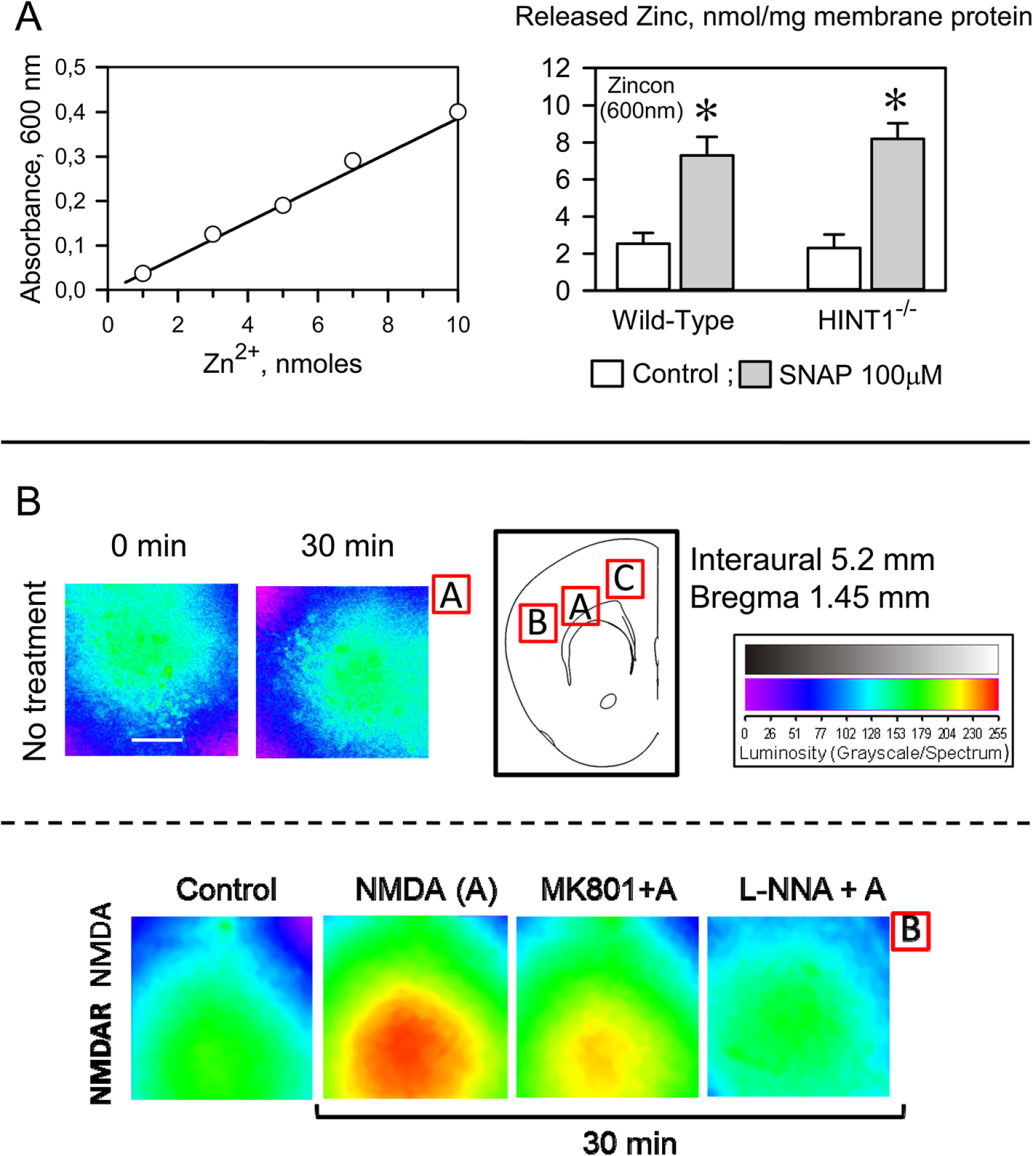
**NMDAR-mediated nNOS/NO activation and zinc release. A**: SNAP release of endogenous zinc from cortical synaptosome membranes obtained from wild-type and HINT1^-/-^ mice. SNAP (100 μM) was added to cortical membranes and the assay was carried out at RT, as described [30]. Left panel: Calibration curve for Zincon detection of Zn^2+^. Right panel: The NO donor was incubated with the membranes for 30 min and Zinc release was monitored by its complexing with the reporter (zinc chelator, Zincon). The absorbance at 600 nm was recorded at RT on a BioChrom Ultrospec 2100 spectrophotometer (Cambridge, UK) and the data represent the mean ± SEM of three independent assays. *Significantly different from the respective control group (without SNAP), *p*<0.05. SNAP produced comparable release of zinc ions from wild-type and HINT1^-/-^ synaptosome membranes. **B**: NMDAR-mediated production of NO and the subsequent release of zinc ions from endogenous stores. The spontaneous endogenous zinc release was determined in Control (untreated) sections. The data shown were obtained at 30 min post-treatment. The effect of NMDAR activation on zinc mobilization was then studied. Control: baseline vehicle; A stands for the agonist NMDA used at 3 μM; MK801+A = 3 μM MK801 (NMDAR antagonist) + 3 μM NMDA; L-NNA+A = 10 μM L-NNA (NOS antagonist) + 3 μM NMDA. The images were color indexed and presented in pseudocolor.

**Figure 5:**
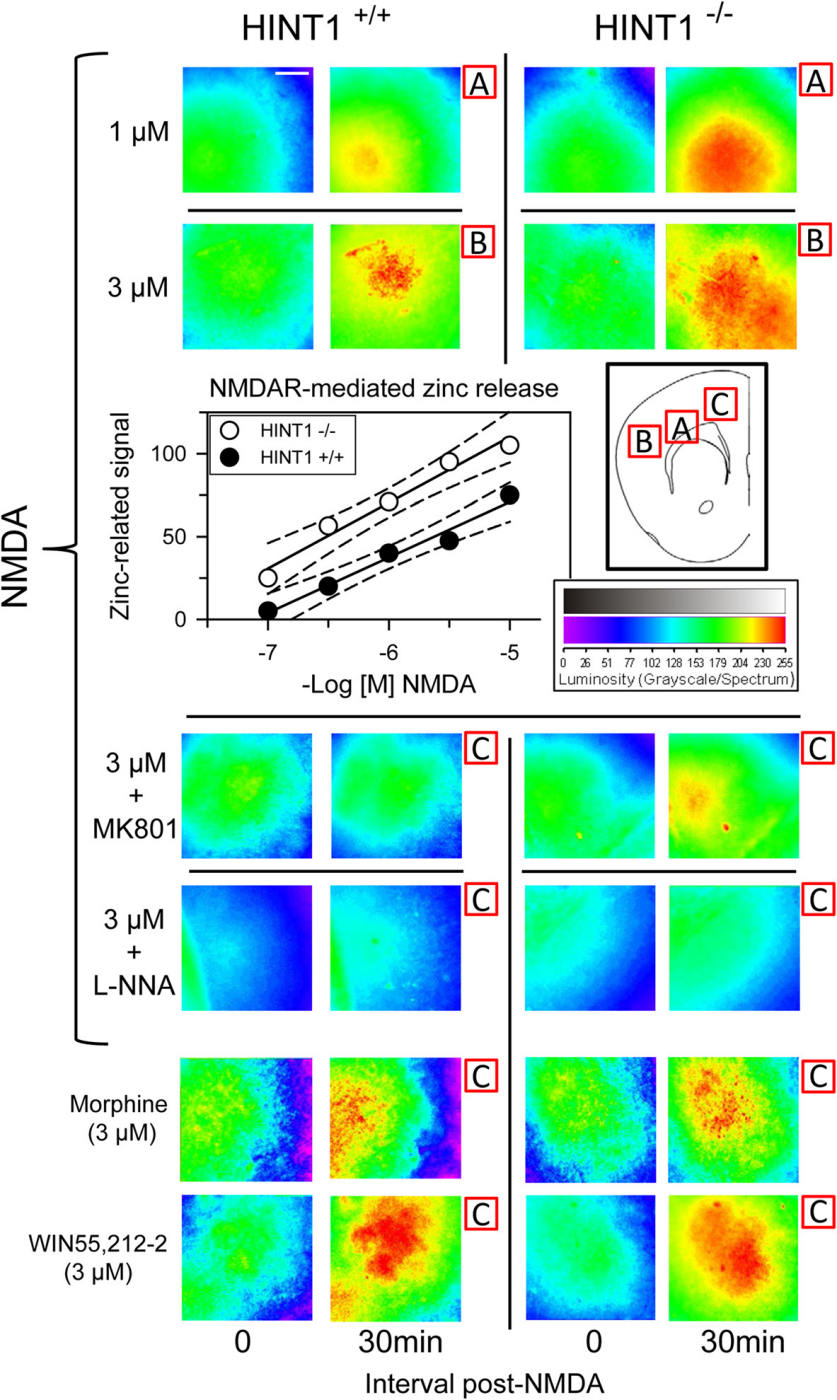
**The absence of HINT1 increases NMDAR-mediated NO production and zinc mobilization in response to NMDA.** Coronal mouse frontal cortex slices from WT and HINT1^-/-^ mice were preloaded with Newport Green diacetate, and fluorescent images were obtained using a 10 × 0.4 HC PL APO objective (excitation, 488; emission, 498–520). The cortical regions studied are indicated as A, B or C, and the data shown were obtained 30 min post-treatment. NMDA was used at the concentrations indicated in the inset, and images for 1 μM and 3 μM are shown. Morphine and WIN55,212-2 were used at 3 μM. The NMDAR antagonist MK801 was used at 3 μM, and the NOS inhibitor L-NNA was used at 10 μM. For each treatment, the assays for wild-type (WT) and HINT1^-/-^ cortical slices were performed during the same run, and the images obtained were color-indexed and presented in pseudocolor [31]. Scale bar = 500 μm. The assay was typically repeated 3 times, and the results were always comparable. Representative images are shown. Inset: Linear regression and 95% confidence intervals of the zinc release promoted by increasing concentrations of NMDA in cortical slices from WT and HINT1^-/-^ mice.

The function of NMDARs is linked to the activation of nNOS and the NO-mediated regulation of zinc metabolism (Figure 4) [5]. Certainly, the de-regulation of zinc homeostasis has been shown to contribute to neurotoxicity [45]. As the absence of HINT1 reduced cell viability in response to NMDA insult, we explored the possibility that NMDAR-mediated NO production and zinc release were regulated by HINT1. We first determined whether the endogenous content of zinc ions (both free ions plus those complexed to proteins) was comparable in membranes of cortical synaptosomes obtained from wild-type and HINT1^-/-^ mice (Figure 4A). Then, we evaluated the release of endogenous zinc in response to NMDAR activation. The incubation of mouse brain cortical slices with NMDA increased Newport Green fluorescence, a measure of zinc ion release from endogenous stores via nNOS/NO in response to NMDAR activity. NMDAR-mediated zinc release was attenuated by MK801 and prevented by NOS inhibition (Figure 4B). However, NMDA evoked a significantly greater mobilization of zinc ions in the absence of HINT1. This enhancement was not observed for morphine or WIN55,212-2, agonists of the MOR and CNR1, suggesting that HINT1 regulates the activity of NMDARs but not that of GPCRs (Figure 5).

**Figure 6:**
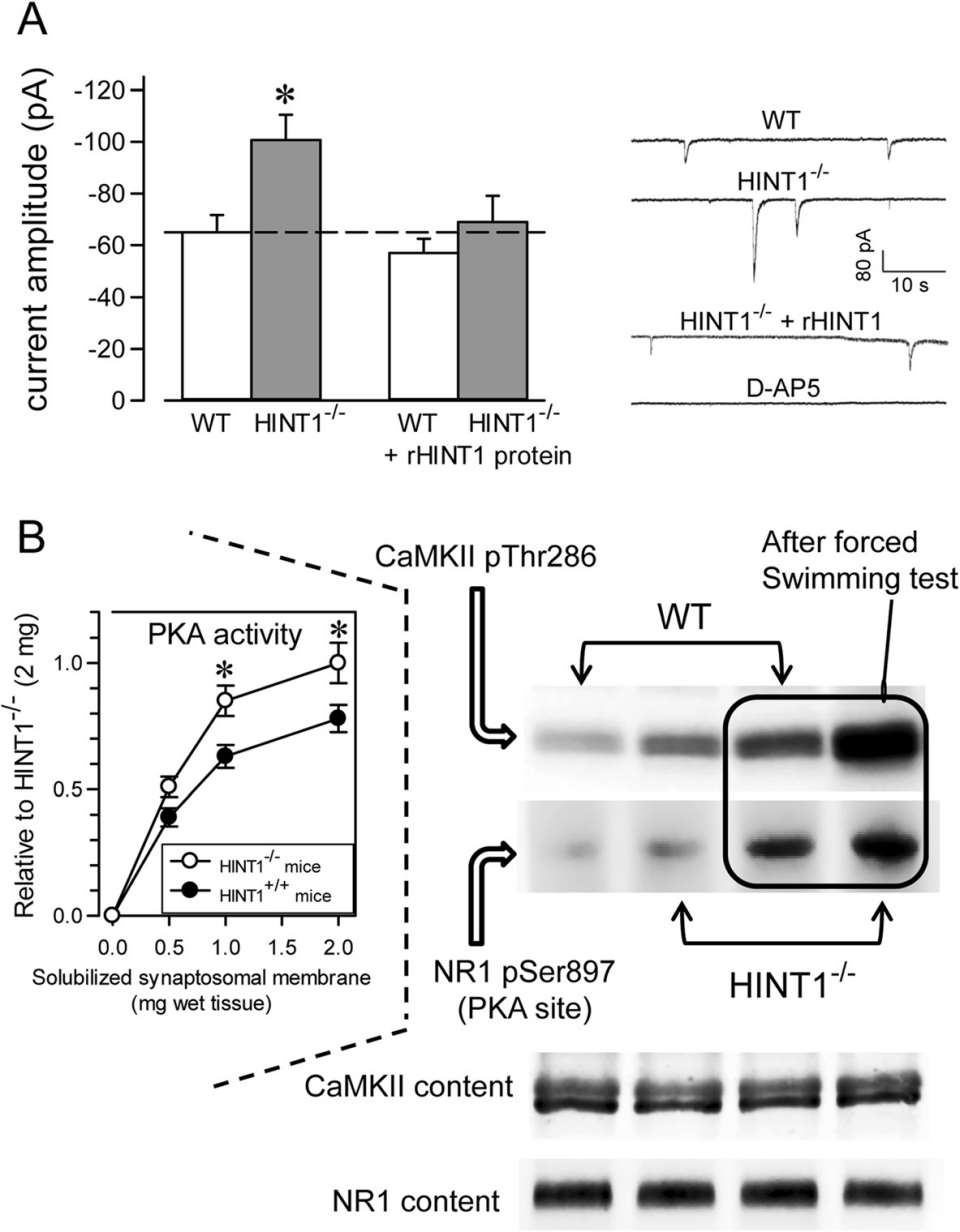
**The effects of HINT1 on the responsiveness of NMDAR. (A)** Spontaneous NMDAR-mediated currents in CA1 pyramidal neurons from WT and HINT1^-/-^ mice. The mean amplitudes of the spontaneous, NMDAR-mediated currents recorded in neurons from WT and HINT1^-/-^ mice in control conditions and when HINT1 was included in the solution of the recording pipette. The data are represented as the mean ± S.E.M. from WT (n = 12) and HINT1^-/-^ (n = 28) mice. * Significant difference *p* < 0.05. Representative whole-cell currents recorded from CA1 pyramidal neurons from WT and HINT1^-/-^ mice with the internal control solution and neurons recorded with HINT1 added to the internal solution. Higher amplitudes of the NMDAR-mediated slow inward currents were observed in HINT1^-/-^ mice relative to WT samples, which reverted in the presence of HINT1. The NMDAR antagonist D-AP5 (50 μM) abolished these currents. **(B)** The effect of mild stress induced by the forced swimming test on NMDAR activity in WT and HINT1^-/-^ mice. Thr286 CaMKII autophosphorylation and PKA-mediated Ser897 NR1 phosphorylation were measured in the controls and mice exposed to the test. Inset: PKA activity of cortical synaptosomes in WT and HINT1^-/-^ mice.

To further investigate the effects of HINT1 on NMDAR function, whole-cell currents from CA1 pyramidal neurons were recorded in hippocampal slices from HINT1^+/+^ and HINT1^-/-^ mice. Whereas the activity of synaptic NMDARs was only slightly increased in the preparations from HINT1^-/-^ mice, the amplitude of the spontaneous slow inward currents that are selectively mediated by the activation of extrasynaptic NMDA receptors was significantly higher in HINT1^-/-^ mice than in WT mice (mean amplitudes: -100.7 ± 7.8 pA, n = 60 and -64 ± 5.7 pA, n = 40; in 10 and 8 neurons from HINT1 ^-/-^ and HINT1^+/+^ mice, respectively). The identity of these NMDAR currents was confirmed by their sensitivity to the NMDAR antagonist D-AP5 [46-48]. To test whether these differences were specifically due to HINT1, we recorded the NMDAR-mediated currents using purified protein in the whole-cell recording pipette. The presence of HINT1 in the intracellular solution did not affect the WT neurons but reversed the changes observed in the transgenic mice (mean amplitude: -70.6 ± 8.1 pA, n = 67, from 8 neurons) (Figure 6A).

Compared to the HINT1^+/+^ controls, the HINT1^-/-^ mice demonstrated increased markers of NMDAR function, such as CaMKII pThr286 autophosphorylation [49] and the phosphorylation of NR1 subunits on Ser897 [3], which are both facilitated by PKA activity (Figure 6B). Similarly, compared to the WT littermates, the HINT1^-/-^ mice displayed higher PKA activity and greater PKA-induced CaMKII activation and NR1 C1 Ser897 phosphorylation in response to stress induced by the forced swimming test (Figure 6B). Thus, HINT1 negatively regulates NMDAR function and is neuroprotective against NMDA-induced excitotoxicity.

### GPCRs diminish NMDAR responsiveness via HINT1

**Figure 7:**
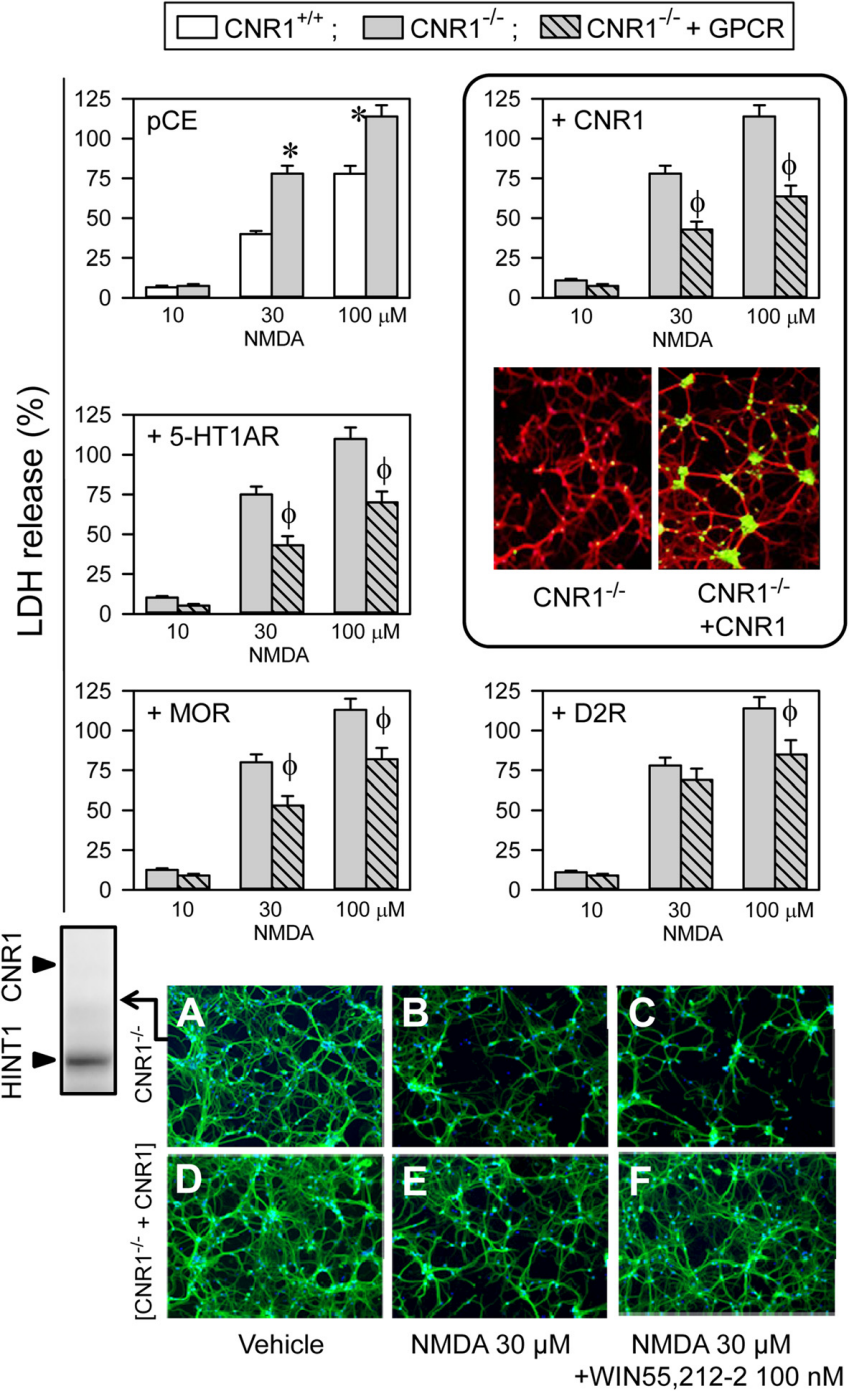
**The expression of GPCRs reduces NMDAR activity.** Cortical cell cultures from WT and CNR1^-/-^ mice were exposed to NMDA for 24 h. * Significant difference compared to WT cultured neurons, *p* < 0.05. Cell death induced by NMDA was also determined in cultures transfected with CNR1, serotonin receptor type 1A (5HT1AR), Mu-opioid receptor (MOR) and dopaminergic receptor type 2 (D2R). Φ Significant difference in the CNR1^-/-^ cultured neurons that were not transfected with the corresponding GPCR, *p* < 0.05. The data shown represent the mean ± S.E.M. from 12 wells per experimental group. Inset to CNR1 group: Fluorescence photomicrograph of CNR1^-/-^ cortical cell cultures showing the expression of transfected CNR1 using Abs against CNR1 (green) and MAP2 (red). The nuclei were counterstained with 4,6-diamidino-2-phenylindole. Bottom: The cells were immunolabelled with an anti-MAP2 Ab (green) and assayed following treatment with vehicle **(A** and **D)**, 30 μM of NMDA **(B** and **E)** or 30 μM of NMDA plus 100 nM of WIN55,212-2 **(C** and **F)**. The expression of HINT1 in the CNR1^-/-^ cortical cell cultures is shown.

HINT1 is present in cortical cultures from mice deficient in CNR1 (CNR1^-/-^), and these cells were more sensitive to NMDA toxicity than cells from WT mice [42] (Figure 7). As the HINT1 protein interacts with the cytosolic regions of different GPCRs, including the CNR1, MOR, serotonin 5HT1A receptor and the dopamine D2 receptor [5-7,31], we determined whether these GPCRs might assist HINT1 to negatively regulate NMDAR function. With the exception of the CNR1, most GPCRs are absent from neurons in primary cultures that are established at this stage of ontogenesis (from E16 embryos), including the dopamine D2 receptors, serotonin 5HT1/2 receptors and MORs [50-52], and thus we forced their expression in these cells. Our results indicated that CNR1^-/-^ cortical cultures transfected with CNR1 receptors were much less sensitive to NMDA insult (Figure 7A,B vs D,E), and the expression of GPCRs in the CNR1^-/-^ cultured neurons during this stage of neural development also led to reduced NMDA toxicity. Thus, 5HT1A receptors and MOR significantly reduced NMDAR excitability. The D2Rs had the weakest effect on NMDAR activity (Figure 7), and this effect of the GPCRs was not modified by their respective antagonists (NMDA 30 μM, antagonists 10 μM and % LDH release as mean ± SEM), WAY100135 (5HT1AR) 42 ± 4, naltrexone (MOR) 48 ± 5, L-741,626 (D2R) 70 ± 6, SR141716A (CNR1) 42 ± 5, n=3). CNR1 agonists, such as WIN55,212-2, when used in the nM range were protective against NMDA excitotoxicity (Figure 1B). However, 1 μM concentrations of the other GPCR agonists failed to provide protection (DPAT (5HT1AR) 49 ± 4, morphine (MOR) 59 ± 5, or quinpirole (D2R) 71 ± 6, n=3).

### HINT1 facilitates the association between MOR/CNR1 and NR1 subunits

**Figure 8:**
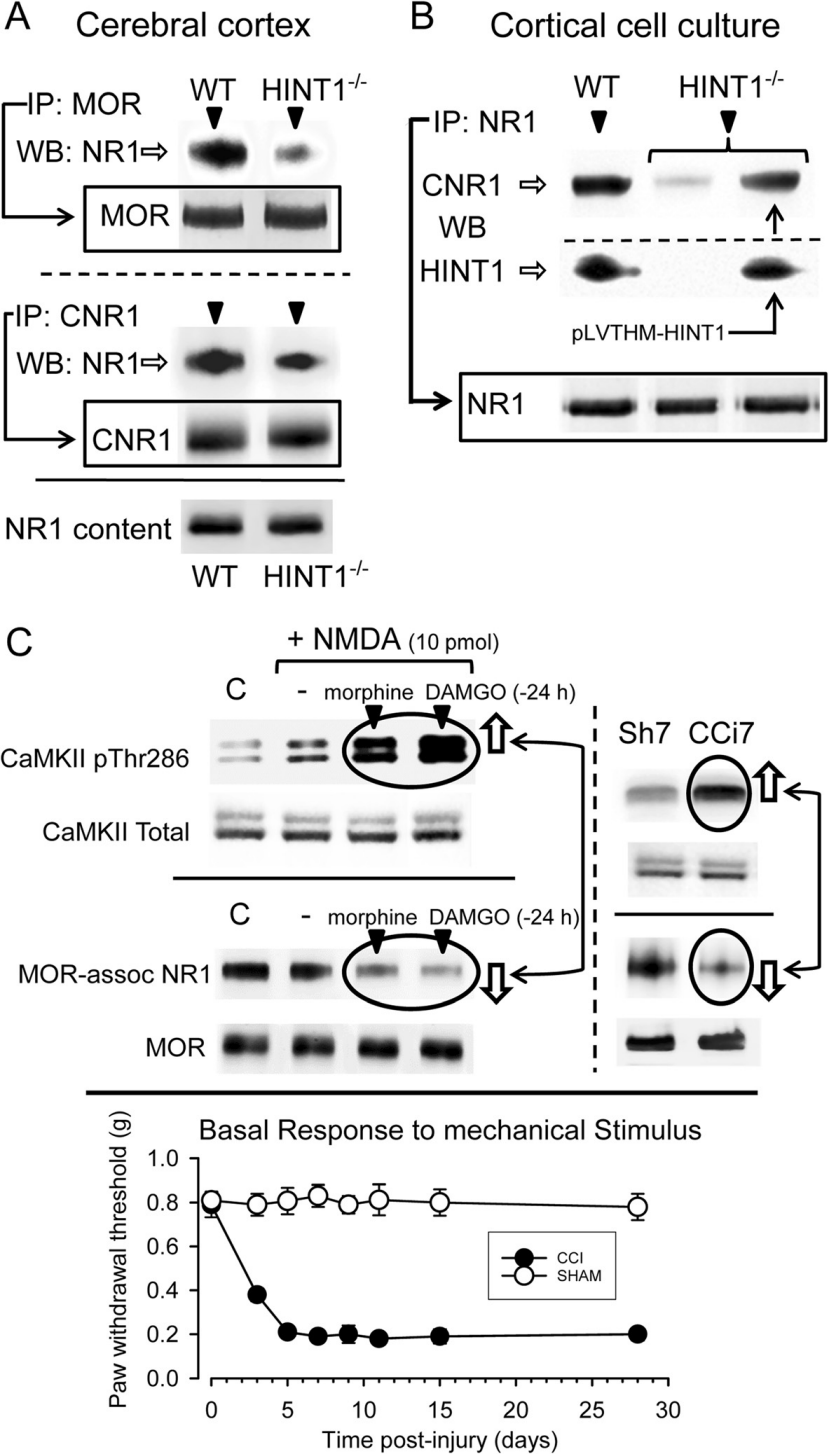
**MOR and CNR1 associate with the NR1 subunits via HINT1: Implications for NMDAR activity. (A)** The association of GPCRs, MOR and CNR1 with the NMDAR NR1 subunits is dependent on HINT1. The GPCRs were immunoprecipitated from solubilized mouse brain cortical synaptosomes from WT and HINT1^-/-^ mice, and the proteins that co-precipitated with the NR1 subunits were analyzed by western blot. **(B)** The association of CNR1 with NR1 in WT and HINT1^-/-^ cortical cell cultures uninfected or infected with 10 μL/well of HINT1^-/-^ lentiviral particles. The presence/absence of HINT1 was determined, and the co-precipitates of the NR1 subunits and CNR1 were then assessed by immunoprecipitation and western blot. For each determination, cells from 10 wells were pooled, and the assay was repeated twice with identical results. **(C)** Opioid agonists promoted a higher level of responsiveness of the NMDAR by inhibiting the association between MOR and NR1. Morphine or DAMGO was icv-injected into the mice 24 h prior to the analysis of MOR/NMDA NR1 association and CaMKII activating autophosphorylation on Thr286. At 7 days post-surgery, the mice from the CCI neuropathic pain group displayed increased pThr286 CaMKII and reduced MOR-NR1 association compared to the sham-operated controls. *Changes of Mechanical Withdrawal Threshold*. Following ligation, animals developed significant mechanical allodynia by day 3 that remained until day 21. Naïve control and sham – operated mice failed to exhibit mechanical allodynia. Data are mean ± SEM. *P<0.05 vs. sham-operated group, ANOVA, followed by the Student-Newman-Keuls test (SigmaStat, SPSS Science Software, Erkrath, Germany) p < 0.05.

MOR and CNR1 associate with NR1 subunits of NMDARs [3,5], and the HINT1 protein stabilizes their interaction [5,53]. In synaptosomes from the adult mouse cerebral cortex, both MOR and CNR1 co-precipitated with the NR1 subunits, and this association was much weaker in HINT1^-/-^ mice (Figure 8A). In cortical cultures from WT mice, the NR1 subunits co-precipitated with CNR1 and HINT1. In HINT1^-/-^ neurons, the amounts of the NR1 subunits and CNR1 were similar to those in HINT1^+/+^ neurons. However, in the absence of HINT1, much less CNR1 co-precipitated with the NR1 subunits compared to the WT cultures. Furthermore, infection of HINT1^-/-^ neurons with HINT1 lentiviral particles increased HINT1 expression, which resulted in the co-precipitation of CNR1 with the NR1 subunits (Figure 8B).

Our results further demonstrated that in WT HINT1^+/+^ cells, the presence of certain GPCRs was sufficient to reduce the impact of NMDA insult and neuronal death (Figure 7). This effect was related to the association of these receptors (MOR and CNR1 with the NR1 subunits) via HINT1. Thus, we next investigated whether the dissociation of NMDARs from MORs control would lead to an increased responsiveness to their activators, which was observed in cultured neurons in the absence of HINT1 or CNR1. Opioids, such as morphine and DAMGO, promote PKC-mediated dissociation of MOR from NR1 subunits [3]. In these circumstances, intracerebroventricular (icv) injection of NMDA to WT mice activated the NMDAR effector CaMKII to a much greater extent than in mice that were not treated with these opioids and showed MOR-NR1 association (Figure 8C).

Neuropathic pain is accompanied with an excess of glutamate activity and the overactivation of NMDARs [8]. Thus, we studied the stability of the MOR-NMDAR complex in mice suffering from chronic constriction injury (CCI), a model of neuropathic pain, for 7 days. We found that the activity of the NMDAR/CaMKII pathway was enhanced, and MOR was not associated with the NR1 subunits (Figure 8C).

## Discussion

Our study showed that HINT1 inhibits the responsiveness of glutamate NMDARs to exogenous and endogenous activators when released during a mild stress response. This inhibition seems to be mediated by the interaction of HINT1 proteins with NMDAR NR1 subunits and requires the cooperation of non-activated GPCRs, such as CNR1, MOR or 5-HT1AR. The absence of HINT1 weakened the association between NMDAR NR1 subunits and GPCRs, enhancing the responses of NMDARs to activators. The activation of most GPCRs promotes that of NMDARs and for the MOR, this is accompanied by the separation of MOR-HINT1 complex from the NMDAR NR1 subunit. Therefore, several GPCRs restrain the activity of NMDARs, although this control can be released in response to their activation, thereby contributing to NMDAR signaling. By contrast, the activated CNR1 maintains a negative influence on NMDAR gating, which facilitates its protective effect against NMDA excitotoxicity. Thus, the HINT1 protein emerges as an essential regulator of these GPCR-NMDAR interactions.

Synaptic NMDARs are mostly targeted to the postsynaptic region of glutamatergic synapses, where they structurally organize (and spatially restricted) into large macromolecular signaling complexes composed of scaffolding and adaptor proteins. These structures physically link the NMDAR to kinases, phosphatases, GPCRs and other signaling molecules [54,55]. Moreover, these interactions between GPCRs and NMDARs may occur more frequently than suspected, and physical interactions between the dopamine D1 receptor, group I metabotropic glutamate receptor (mGlu5a), MOR or CNR1 and the C1 segment of NMDAR NR1 subunits have also been reported [3,5,56,57].

In brain synaptosomes from HINT1^-/-^ mice, the quantities of the NR1 subunits, CNR1, MOR and zinc content were similar to those in WT mice. However, in the absence of HINT1 the association between these GPCRs and the NR1 subunits weakened, and consequently, NMDARs mobilized more zinc ions from endogenous stores than in the WT controls. Thus, HINT1 stabilizes the coupling of CNR1s with NMDAR NR1 subunits that is required for cannabinoids to dampen NMDAR activity. Indeed, in HINT1^-/-^ cortical neurons NMDAR responsiveness is enhanced and the presence of CNR1 was not sufficient to inhibit this activity, with WIN55,212-2 failing to protect against cell death upon a NMDA insult [5]. In electrophysiological studies, hippocampal preparations from HINT1^-/-^ mice exhibit increased extrasynaptic NMDAR current activity. Whereas synaptic NMDARs contribute to neuroprotection, stimulation of extrasynaptic NMDARs promotes cell death and a perturbation in this balance is currently believed to contribute to the etiology of neurodegenerative diseases [58]. Mood disorders, like mania, concur with altered NMDAR function, and bipolar disorder patients have fewer glial and neuronal cells [59,60]. Interestingly, when evaluated in the battery of conventional behavioral tests, HINT1^-/-^ mice displayed manic-like behavior [61].

The activation of several GPCRs triggers that of the associated NMDARs via PKC/Src, a signaling mechanism that in certain situations negatively influences the activity of the GPCR itself, as described for the MOR [33]. The activity of the NMDAR within this regulatory loop must be tightly controlled to prevent the consequences of its de-regulation. By contrast, it is the activity of the NMDARs themselves that makes the demands on the endogenous cannabinoid system to control their calcium currents [62]. Therefore, the association of CNR1s with NR1 subunits through HINT1 proteins persists during NMDAR activation, and cannabinoids then exert negative control on the NMDARs’ activity [5]. The differences that GPCRs display in the regulation of NMDARs is apparently unrelated to the class of G proteins they regulate, since while D1 regulates Gs/Gq [63], the MOR, CNR1, D2R and 5HT1A regulate Gi/o, and Gq couples to MOR, CNR1, 5HT2A [18,64-66]. It is possible that the association of the HINT1 protein with the GPCR determines the relation of the latter with the NMDAR. Several GPCRs bind the HINT1 protein through their cytosolic regions, such as the MOR, CNR1, 5HT1AR, 5HT2AR, muscarinic M2 and M4 receptors, α2 Adrenergic receptor, dopamine D2 receptor [31], D1 receptor (unpublished observation), mostly through the C terminal or third internal loop. Under physiological conditions, the resting CNR1 (as well as GPCRs like the MOR or the 5HT1A receptor) interact with HINT1 proteins to downregulate the activity of NMDARs, thereby restraining the overall NMDAR currents that could otherwise promote irreversible neuronal damage. Thus, in the context of NMDAR regulation by GPCRs, HINT1 emerges as an essential protein to protect against neuronal damage.

We have characterized the dynamics of MOR-HINT1 and CNR1-HINT1 associations with NMDARs in terms of receptor activation, identifying critical differences [5,6]. Opioids like morphine activate PKC, which disrupts the association of MOR-HINT1 with NMDAR NR1 subunits. However, when activated NMDARs recruit CaMKII activity, this increases their association with CNR1s and this is a relationship that is then further enhanced by PKC. These kinases remove certain proteins, probably RGS-Rz proteins [6], from the CNR1-bound HINT1 protein, thereby promoting its binding to NR1 subunits. CaMKII plays different roles in CNR1 and MOR regulation, and whereas this kinase activity is implicated in MOR desensitization [67], in the CNR1-HINT1 environment it prevents the action of PKC on the NR1 C1 segment that would separate both receptors. Those GPCRs that enhance NMDAR function through the activation of PKC, intracellular Ca2+ release and Src activation, also promote serine phosphorylation of NR1 C1 [1,4,68], comparable to that which occurs with the MOR. For other receptors that couple to and inhibit NMDARs, it would be necessary to address the molecular mechanisms behind their effects and to compare them with those of CNR1s.

The complex GPCR-HINT1 can interact with the C1 segment of NMDAR NR1 subunits [5] and this interaction reduces the gating of the tretrameric ionotropic receptor. The regulation of calcium fluxes permeating the NMDAR is complex and results from an interplay between different systems. Glutamate, glycine and D-serine bind to the extracellular domains of the NR1 and NR2/3 subunits, which augments calcium transit. Zinc ions co-released with glutamate reach micromolar concentrations in the cleft and they diminish the responsiveness of NMDARs to activation [69], these zinc ions displaying a higher affinity for NR2A subunits than NR2B subunits [70]. The sensitivity of the NMDAR to extracellular stimuli is regulated by the serine, threonine and tyrosine phosphorylation/dephosphorylation of cytosolic residues on the NR1/2 subunits [2,71]. By contrasts, PKC phosphorylates the NR1 subunits, which blocks the binding of negative regulators [72,73], and Src acts on the NR2A/B subunits to remove the tonic inhibition of extracellular zinc [74], resulting in an increase in the entry of extracellular calcium. The protein phosphatase calcineurin reverses the phosphorylation of the regulatory C1 region of the NR1 subunits and it plays an important role in the inactivation of NMDARs [75], probably favoring the binding of negative regulators. Moreover, the striatal-enriched protein tyrosine phosphatase (STEP) reverses the Src-mediated upregulation of NMDAR activity [76].

The control of NMDAR function is also achieved by direct physical interactions with third party proteins like calmodulin (CaM) and probably GPCR-HINT1. The calcium-dependent inactivation (CDI) of NMDARs is a negative feedback response that downregulates the gating of NMDAR channels and the calcium-binding protein CaM plays a key role in this response. The cytoskeletal protein α-actinin2 interacts with the C0 region of the NR1 subunits and calcium-activated CaM promotes CDI by releasing the NMDAR complex from the cytoskeleton. The NR1 C1 segment binds Ca^2+^-CaM rather than CaM, and this binding reduces the “open” probability of the NMDAR-channel [77-79]. In addition, GPCR-activated PKC phosphorylates the C1 region at Ser890 and Ser896, and it blocks Ca^2+^-CaM binding [80,81], thereby promoting NMDAR activity. Thus, when coupled to non-activated GPCRs HINT1 binds to this C1 region of the NR1 subunits and like Ca^2+^-CaM it inhibits NMDAR function [5] present study. In this context, the activation of GPCRs like the MOR and mGlu1α enhances NMDAR activity in a PKC-dependent manner, which releases GPCR-HINT1 inhibition and prevents the binding of Ca^2+^-CaM by acting on serines in the NR1 C1 domain, and both these effects increase the “open” probability of the calcium channel [3,4,80].

In the CCI animal model of neuropathic pain in which NMDARs are more sensitive to glutamatergic activation, these receptors were separated from the MOR and then released from the control of HINT1. An interesting observation is that cerebral ischemia induces the accumulation of dopamine, serotonin and other neurotransmitters that contribute to neuronal death [82]. Accordingly, MOR antagonists, dopaminergic or serotonergic nerve depletors and glutamate antagonists prevent or reduce the brain injury that results from experimental heatstroke [83,84]. While antagonists targeting MOR or 5HT2AR protect against heatstroke, agonists of CNR1, the delta opioid receptor (DOR), 5HT1AR or mGluR7 also appear to be beneficial [85-87]. These observations suggest that in the absence of agonists, GPCRs can diminish NMDAR calcium fluxes via HINT1. However, in function of the GPCR, its activation can either enhance NMDAR responsiveness or maintain the inhibition of NMDAR function. CNR1 is highly efficacious in counteracting the neuronal damage resulting from NMDAR overactivation, although other GPCRs like DOR, 5HT1AR and mGluR7, could also potentially provide neuroprotection.

## Conclusions

The GPCRs CNR1 and MOR negatively regulate NMDAR responsiveness by interacting with the HINT1 protein. While the control of NMDAR function can be achieved without GPCR activation, many of these agonists were shown to disrupt the GPCR-HINT1 association with NR1 subunits and increase NMDAR responsiveness to activation. Notwithstanding, CNR1 cannabinoids counteracted the negative effects of NMDAR-mediated excitotoxicity and preserved cell viability. As several GPCRs show functional cross-talk with ion channels these interactions might support therapeutic and also undesirable effects of available drugs [88]. Thus, elucidating the role of HINT1 in GPCR regulation of glutamate NMDAR activity will improve our understanding of the mechanisms behind NMDAR-mediated neuronal damage and may provide novel therapeutic targets.

## Methods

### Animal studies

A mouse knock-out strain, 96% genetic background from 129 mice, with targeted disruption of HINT1 (a gift from I.B. Weinstein/J.B. Wang) and wild-type littermate mice were used for this study. Genotyping was performed on the basis of the protocol described previously [6]. All the procedures for handling and sacrificing animals followed the European Commission guidelines (Council Directive 86/609/EEC) and approved by the Committee on Animal Care at CSIC. NMDA was administered in vivo as described previously [3].

Forced swim test: The test was based on the original version of the forced swim test of Porsolt for mice [89]. Mice were placed in a 5 L cylinder (40 cm high, 25 cm diameter) filled with 3.5 L of water, where they swam without the possibility to touch the bottom. Mice were placed in water through a series of four trials of 6 min each and immobility was recorded during the last 4 min of each trial using a stopwatch. Immobility was determined when the mouse was only making movements necessary to keep its head above the water and maintained a stationary posture; a stopwatch was started within the first 2 sec immobility was observed.

Chronic constriction injury (CCI): After testing mice for their basal mechanical sensitivity, CCI was performed under isoflurane/oxygen anesthesia using a modification of the Bennett and Xie procedure [90]. Briefly, the sciatic nerve was exposed at the mid-thigh level, proximal to its trifurcation, and two 5/0 braided silk suture ligatures (Lorca Marin, Murcia, Spain, 70014) were tied loosely around the sciatic nerve, 1–2 mm apart. Sham CCI surgery was carried out identically, except that no ligations were placed around the nerve.

### Primary cortical cell culture

Neuron-enriched mouse cerebral cortical cultures were prepared from the brains of embryonic day-16 wild-type 129 and HINT1 knockout mice. Cerebral cortices were dissociated and seeded (1.25 × 10^5^ cells/cm2) onto multiwell dishes coated with poly-D-lysine. After 3 hours, the culture medium was changed to Neurobasal medium supplemented with B-27, GlutaMAX and antibiotics (100 IU/mL Penicillin and 100 μg/mL Streptomycin solution) (Invitrogen, Paisley, UK). From days 5 to 7 in vitro, cytosine arabinoside (5 μM) was added to the cultures to eliminate the majority of proliferating non-neuronal cells. Cultures were maintained at 37°C in a humidified 5% CO2 incubator. In some cases, cells were evaluated after transfection for 72 h, with the concentrated lentiviral vector coding the HINT1 protein cDNA. In a set of experiments, cultures from CNR1 receptor knockout mice, generously donated by Dr. A. Araque [91], were used.

### Measurement of cell death

Between days 12 and 14 in vitro, cultures were rinsed with serum-free minimal essential medium and treated for 24 h with NMDA, with or without other drugs. Cell death was quantified by measuring lactate dehydrogenase (LDH, Roche) release into the bathing medium over 24 h and was expressed as a percentage of cell death induced by a maximally cytotoxic concentration (500 μM) of NMDA: (LDH - LDHcontrol)/(LDHNMDA - LDHcontrol) × 100%.

### Immunocytochemistry

Cells plated onto poly-D-lysine coated 10 mm-glass coverslips were fixed in 4% paraformaldehyde for 10 min, incubated in 10% normal goat serum (NGS) and 0.1% Triton X-100 in phosphate buffer saline (PBS). The cells were immunolabeled with MAP2ab (M1406, Sigma-Aldrich, Madrid, Spain) and for CNR1 receptor (10006590, Cayman Chemical, Ann Arbor, Michigan) for 2h at room temperature. The cells were then incubated with Alexa fluor 488 or 594 conjugated secondary antibodies (Invitrogen) and finally with 4,6-diamidino-2-phenylindole (DAPI), before mounting in Mowiol solution (Calbiochem, San Diego, CA). Slides were observed with a Leica DMI 6000 inverted fluorescence microscope (Leica Microsistemas S.L.U., Barcelona, Spain). Controls were performed to confirm the specificity of the primary and secondary antibodies.

### Lentiviral vector production

RNAs isolated from mouse brain lysates were reverse-transcribed using the SuperScript^®^ III First-Strand Synthesis System (Invitrogen) following the manufacturer’s instructions. The cDNAs for the murine HINT1 (NM_008248) was then amplified by PCR and subsequently cloned in the pLVTHM “Tet on” inducible vector downstream of the H1 promoter. Cloned inserts were sequenced to verify the integrity of each construct.

Lentiviruses were prepared by cotransfecting 10 μg pLVTHM vector (carrying either HINT1 cDNAs or the empty plasmid), 6.5 μg second generation packaging plasmid (psPAX2) and 3.5 μg envelope plasmid (pMD2.G) into HEK-293T cells. Transfections were carried out with a 1:3 volumetric mix of DNA and FuGENE^®^ 6 Transfection Reagent (Roche). Lentivirus-containing supernatants were collected 48 and 72 h after transfection, filtered through 0.22-μm-pore nitrocellulose, concentrated by ultracentrifugation, aliquoted, and stored at - 80°C until used. The titer of lentivirus was determined by hole-by-dilution titer assay.

### Hippocampal slice preparation and electrophysiology

Cortical hippocampal slices were obtained from wild-type 129 and HINT1 knockout mice (13–15 days old). Animals were decapitated and brains were rapidly removed and placed in ice-cold artificial cerebrospinal fluid containing (in mM): 124 NaCl, 2.7 KCl, 1.25 KH_2_PO_4_, 2 MgSO_4_, 26 NaHCO_3_, 10 glucose, 0.4 ascorbic acid and 2 CaCl_2_, and gassed with 95% O2/5% CO2 (pH 7.3). Slices were incubated for >45 minutes at room temperature and then transferred to an immersion recording chamber and superfused with Mg^2+^-free ice-cold artificial cerebrospinal fluid (ACSF) containing (in mM): 124 NaCl, 2.7 KCl, 1.25 KH_2_PO_4_, 26 NaHCO_3_, 10 glucose, 4 CaCl_2_, 0.01 glycine, 0.05 picrotoxin (to block GABA A receptors) and gassed with 95% O2/5% CO2 (pH 7.3). Extracellular Mg^2+^ was omitted to maximize NMDAR-mediated currents. Cells were visualized under an Olympus BX50WI microscope equipped with infrared and differential interference contrast imaging devices, and with a 40x water-immersion objective.

Electrophysiological recordings from CA1 pyramidal neurons were made using the whole-cell patch-clamp technique with an internal solution containing (in mM): 135 KGluconate, 10 KCl, 10 HEPES, 1 MgCl_2_ and 2 ATP-Na_2_ (pH 7.4). Neurons were recorded in voltage-clamp conditions with the membrane potential held at – 70 mV. NMDA receptor-mediated currents were isolated in the presence of 6-Cyano-7-nitroquinoxaline-2,3-dione (CNQX; to block AMPA-kainate receptors; 20 μM). When indicated, the purified r-HINT1 protein was included in the internal solution at a final concentration of 150 nM. Signals were fed to a Pentium-based PC through a DigiData 1440A interface board. Signals were filtered at 1 KHz and acquired at 10 KHz sampling rate. The pCLAMP 10.2 (Axon instruments, Sunnyvale, CA) software was used for data acquisition and storage.

### Immunoprecipitation and Western blotting

Preparation of membrane from cortical cell cultures and immunoprecipitation of NR1 subunits was performed as described previously [3]. The immunocomplexes were recovered and proteins were resolved by SDS/polyacrylamide gel electrophoresis (PAGE). The separated proteins were then transferred onto 0.2 μm polyvinylidene difluoride (PVDF) membranes (BioRad 162–0176), probed with the primary antibodies to HINT1 (Abnova H00003094-A01. Abyntek, Spain) and CNR1 (C terminal sequence 461–472; Cayman Chemical, Mi, USA, 10006590), and detected using secondary antibodies conjugated to horseradish peroxidase. Antibody binding was visualized by chemiluminescence and recorded with a ChemiImager IS-5500 (Alpha Innotech, San Leandro, California). Densitometry was performed using Quantity One Software (BioRad) and expressed as the mean ± SEM of the integrated volume (average optical density of the pixels within the object area/mm2).

### Enzymatic activity

The Pep Tag protein kinase A assay (Promega, Madison, WI, USA) was used for assessing total PKA enzymatic activity according to the manufacturer’s instructions. Briefly, brain lysates were prepared using RIPA buffer at 4°C. The homogenate was then centrifuged at 18,000×*g* for 15 min at 4°C. The supernatants were incubated for 2 min at 30°C and for 30 min at 30°C with the specific substrate in the presence of activating solution. The reaction was stopped at 95°C for 10 min. The PKA-induced phosphorylation specifically changes the net charge of the fluorescent peptide substrate from +1 to -1. Consequently peptides were separated according to their net charges via electrophoresis in 0.8% agarose in 50 mM Tris–HCl (pH 8.0) in a horizontal gel at 100 V for 15 min. The phosphorylated peptide migrated towards the anode and the non-phosphorylated peptide to the cathode.

### Zinc-microfluorescence imaging in mouse cortical slices

Coronal mouse frontal cortex slices (200 μm; 2.50-1.50 mm to bregma) from WT and HINT1^-/-^ mice were oxygenated and preloaded with 50 μM of the cell-permeable Newport Green diacetate (50 μM; N7991, Invitrogen), 0.1% pluronic acid and 0.5% dimethyl sulfoxide for 1h as described elsewhere [30]. The permeated probe remains trapped and that remaining in the extracellular millieu was removed before adding the substances under study and performing Intracellular Zn^2+^ imaging. The effects of the following compounds were assayed: NMDA; the NMDAR antagonist MK801; the NOS/NO inhibitor LNNA (Tocris, UK, 0664). Images were obtained by confocal microscopy through a 10 × 0.4 HC PL APO objective on a Leica DMIII 6000 CS confocal fluorescence microscope equipped with a TCS SP5 scanning laser (excitation, 488 nm; emission, 498–520 nm). The size and resolution of the captured images were identical and before data analysis it was verified that within the area under study variations in pixel luminosity were normally distributed (Systat Software, Inc., Erkrath, Germany). For each concentration of NMDA and animal group (WT and HINT1^-/-^) differences of luminosity means between control and NMDA-treated images were computed (AlphaEase FC Software, San Leandro, CA, USA). The data were analyzed to determine correlation coefficients and 95% of confidence intervals of WT and HINT1^-/-^ groups and plotted (Sigmaplot/Sigmastat v12, Erkrath, Germany). The images were color indexed and presented in pseudocolor [31].

### Spectrophotometric detection of zinc released from cortical synaptosomes

Samples (0.5 mL total volume) were prepared by adding 100 μL of cortical membrane suspension, vehicle or the NO donor (S)-Nitroso-N-acetylpenicillamine (SNAP) to 400 μL of Hepes buffer (25 mM; pH 7.8; treated with Chelex-100 resin (BioRad). The zinc ion releasing effect of SNAP on brain synaptosomes reached a maximum when used at 100 μM for 30 min [30]. Calibration samples were prepared from ZnCl_2_ (100 mM) solution (Sigma 39059) and Hepes buffer. For blanks, the metal solution was substituted with Chelex-100-treated water. Complexation was initiated by the addition of the zinc chelator Zincon (Sigma #96440) (stock solution 1.6 mM in NaOH 1 M) to reach a final concentration of 40 μM. Absorption spectra (600 nm) were recorded after 20 min of sample incubation at room temperature (RT) on a BioChrom Ultrospec 2100 spectrophotometer (Cambridge, UK).

### Drugs and primary antibodies

R(+)-Win 55212 was purchased from Sigma-Aldrich (Madrid, Spain), N-Methyl-d-aspartate (NMDA), Ly320135, JTE907, MK801, LNNA, DAMGO and AP5 were obtained from Tocris (Abingdon, UK). Morphine sulfate was from Merck (Darmstadt, Germany). Recombinant HINT1 protein was obtained as previously described [3,6]. The antibodies used in this study were: MAP2ab (M1406, Sigma-Aldrich, Madrid, Spain), HINT1 (Abnova H00003094-A01, Abyntek, Spain) and CNR1 receptor (10006590, Cayman Chemical, Ann Arbor, Michigan) NMDAR1 (Abcam ab1880); Ca^2+^/calmodulin-dependent protein kinase II (BD 611292); Phospho- Ca^2+^/calmodulin-dependent protein kinase II (CaMKII Thr286, Cell Signaling 3361).

### Data analysis

Data were expressed as mean ± S.E.M. ANOVA, followed by the Student-Newman-Keuls test (SigmaStat, SPSS Science Software, Erkrath, Germany) was performed and significance was defined as *P* < 0.05.

## Abbreviations

CCI: Chronic constriction injury; CNR1: Cannabinoid receptor type 1; GPCRs: G protein-coupled receptors; HINT1: Histidine triad nucleotide-binding protein 1; LDH: Lactate dehydrogenase; MOR: Mu-opioid receptor; NMDA: N-methyl-D-aspartate; PAGE: SDS/polyacrylamide gel electrophoresis; PKC: Protein kinase C; PKCi: Protein kinase C-interacting protein.

## Competing interests

The authors declare that they have no competing interests.

## Authors’ contributions

AVS, PSB and MRM performed the experiments. JG and PSB conceived and designed the study and wrote the paper. All authors read and approved the final manuscript.
